# Expression Patterns of Plasmodium falciparum Clonally Variant Genes at the Onset of a Blood Infection in Malaria-Naive Humans

**DOI:** 10.1128/mBio.01636-21

**Published:** 2021-08-03

**Authors:** Anastasia K. Pickford, Lucas Michel-Todó, Florian Dupuy, Alfredo Mayor, Pedro L. Alonso, Catherine Lavazec, Alfred Cortés

**Affiliations:** a ISGlobal, Hospital Clínic-Universitat de Barcelona, Barcelona, Catalonia, Spain; b INSERM U1016, Centre National de la Recherche Scientifique (CNRS) Unité Mixte de Recherche (UMR) 8104, Université de Paris, Institut Cochin, Paris, France; c Centro de Investigação em Saúde de Manhiça, Manhiça, Mozambique; d Consorcio de Investigación Biomédica en Red de Epidemiología y Salud Pública (CIBERESP), Madrid, Spain; e ICREA, Barcelona, Catalonia, Spain; National Institute of Allergy and Infectious Diseases

**Keywords:** *Plasmodium falciparum*, chromatin, clonally variant genes, controlled human malaria infection, epigenetics, heterochromatin, malaria, transcription

## Abstract

Clonally variant genes (CVGs) play fundamental roles in the adaptation of Plasmodium falciparum to fluctuating conditions of the human host. However, their expression patterns under the natural conditions of the blood circulation have been characterized in detail for only a few specific gene families. Here, we provide a detailed characterization of the complete P. falciparum transcriptome across the full intraerythrocytic development cycle (IDC) at the onset of a blood infection in malaria-naive human volunteers. We found that the vast majority of transcriptional differences between parasites obtained from the volunteers and the parental parasite line maintained in culture occurred in CVGs. In particular, we observed a major increase in the transcript levels of most genes of the *pfmc-2tm* and *gbp* families and of specific genes of other families, such as *phist*, *hyp10*, *rif*, or *stevor*, in addition to previously reported changes in *var* and *clag3* gene expression. Increased transcript levels of individual *pfmc-2tm*, *rif*, and *stevor* genes involved activation in small subsets of parasites. Large transcriptional differences correlated with changes in the distribution of heterochromatin, confirming their epigenetic nature. Furthermore, the similar expression of several CVGs between parasites collected at different time points along the blood infection suggests that the epigenetic memory for multiple CVG families is lost during transmission stages, resulting in a reset of their transcriptional state. Finally, the CVG expression patterns observed in a volunteer likely infected by a single sporozoite suggest that new epigenetic patterns are established during liver stages.

## INTRODUCTION

*Plasmodium* spp. are responsible for the globally important disease malaria, which causes over 200 million clinical cases and almost half a million deaths per year (1). The complex life cycle of malaria parasites is split between different hosts and cell types. The invertebrate mosquito host injects the infective parasite forms known as sporozoites into the vertebrate host during a blood meal. Sporozoites travel to the liver and multiply asexually within hepatocytes, generating merozoites that are then liberated into the bloodstream, where the intraerythrocytic development cycle (IDC) begins. This cycle consists of erythrocyte invasion followed by asexual multiplication, including the ring, trophozoite, and multinucleated schizont stages, and release of new merozoites. Repeated rounds of the IDC enable parasites to rapidly increase their biomass and establish an enduring infection in the vertebrate host. However, some parasites convert into sexual forms termed gametocytes and abandon the IDC. Mature male and female gametocytes are infective to mosquitoes. Mating occurs within the mosquito midgut, and after several complex development steps, new sporozoites are generated, closing the cycle (2).

Life cycle progression in *Plasmodium* spp. is controlled mainly at the transcriptional level such that each stage is characterized by a specific gene expression program (3–5). This enables parasites to thrive in the multiple different environments they are exposed to, which entail dramatic differences in conditions such as temperature, pH, and nutrient availability. However, in addition to environmental diversity associated with life cycle progression, malaria parasites also have to confront fluctuating conditions within the same niche. These fluctuations, which can occur between individual hosts of the same species or even within the course of a single blood infection, may derive, for instance, from changes in the host’s physiological or immunological state or from the effects of antimalarial treatment (6, 7). Adaptation to such diverse conditions occurs through various genetic and nongenetic mechanisms. While in malaria parasites genetic changes play a major role in species evolution and long-term adaptation to new conditions, as in any other organism, rapid adaptation to conditions that fluctuate frequently requires reversible, dynamic mechanisms that provide phenotypic plasticity (i.e., alternative phenotypes from the same genome) (8). One such mechanism is clonally variant gene (CVG) expression, which refers to genes that can be found in a different state (active or silent) in different individual parasites with identical genomes and at the same stage of life cycle progression. While both states are heritable, CVGs undergo low-frequency stochastic switches between the active and silent states, which constantly generates transcriptional heterogeneity within parasite populations (9–11). Changes in the expression of these genes can result in phenotypic variation; therefore, when the conditions of the environment change, natural selection can operate upon this preexisting diversity and eliminate from the population parasites with CVG expression patterns that do not confer sufficient fitness under the new conditions. This is considered a bet-hedging adaptive strategy (12), a type of adaptive strategy commonly observed in many microbial species (13–15).

In P. falciparum, the most virulent human malaria parasite species, CVGs include gene families such as *var*, *rif*, *stevor*, *pfmc-2tm*, *hyp1* to *hyp17*, *phist*, and *surfin* linked to pathogenesis, antigenic variation, and host cell remodeling; *mspdbl2*, *eba140*, and *pfrh4* genes linked to erythrocyte invasion; *clag* genes involved in solute transport; *acs* and *acbp* families linked to acyl-CoA metabolism; and *pfap2-g*, the master regulator of sexual conversion (9–12), among others. Specific adaptive roles have been demonstrated for changes in the expression of *var* (16–18) and *clag3* (19–24) genes.

The active or silenced state of CVGs is regulated at the epigenetic level (25). These genes are mainly located in subtelomeric bistable chromatin domains, in which both the active (euchromatin) and the silenced (facultative heterochromatin) states can be stably transmitted for several generations of asexual growth. Spontaneous transitions between the two states underlie the transcriptional switches (8–11). For all CVGs analyzed so far, the silenced state is characterized by the posttranslational histone modification histone H3 lysine 9 trimethylation (H3K9me3) and heterochromatin protein 1 (HP1), whereas the active state is associated with acetylation of H3K9 (H3K9ac). Transmission of these histone modifications through asexual replication constitutes the epigenetic memory for the transcriptional state of CVGs (8–11, 26–30). In *var* and *clag3* genes, the only P. falciparum genes that are known to show mutually exclusive expression (i.e., only one member of the family is active at a time in individual parasites) (31–35), this epigenetic memory is erased during transmission stages (here including gametocyte, mosquito, and liver stages) (23, 36–38).

The expression patterns of CVGs under culture conditions have been characterized for several specific gene families and also at a genome-wide scale (12, 32, 33, 39, 40). However, understanding how CVGs are expressed under the natural conditions of human infection is complicated by multiple factors, including common occurrence of polyclonal infections and genetic diversity among isolates, which mainly affects CVGs. The expression of only some specific CVG families such as *var*, *clag*, and genes involved in erythrocyte invasion has been characterized in some detail in natural infections (8, 16, 23, 41–43). This is an important gap of knowledge because the conditions of the environment influence the expression patterns of CVGs that prevail. Indeed, the limited data available suggest that the transcriptome of malaria parasites grown *in vitro* differs substantially from that observed *in vivo*, including differences in CVG expression (44–47).

Parasites obtained from controlled human malaria infection (CHMI) trials provide many of the advantages of both cultured parasites and parasites from natural human infections because they are exposed to “real” human host conditions, but they have a well-defined genetic background. Furthermore, the conditions of the host and the time of infection are well controlled, which reduces the number of variables and facilitates the interpretation of the results. Therefore, CHMI trials provide a valuable system to study malaria parasite biology, including *in vivo* expression of CVGs. So far, the analysis of gene expression in CHMI samples has focused mainly on the *var* and *clag* families (23, 36–38, 48–50). Two more recent studies analyzed the transcriptome of parasites obtained from CHMI volunteers at a genome-wide level. However, these studies only included ring-stage parasites, precluding the characterization of transcriptional patterns for genes expressed at other stages of the IDC (51, 52).

To provide a complete view of P. falciparum CVG expression patterns during the initial phase of a blood infection in malaria-naive humans, we performed a genome-wide transcriptomic comparison across the full IDC between parasites obtained from volunteers participating in a CHMI trial and the parental line maintained in culture. With this controlled approach, we identified transcriptional differences between parasites growing under *in vitro* culture conditions or growing in the human circulation after passage through transmission stages. To confirm the epigenetic nature of the differences observed, we mapped the genome-wide distribution of heterochromatin. We also tested the hypothesis that CVGs other than the mutually exclusively expressed *var* and *clag3* genes undergo an epigenetic reset during transmission stages.

## RESULTS

### Transcriptomic comparison between parasites obtained from CHMI volunteers and the parental line reveals changes in CVG expression.

**FIG 1:**
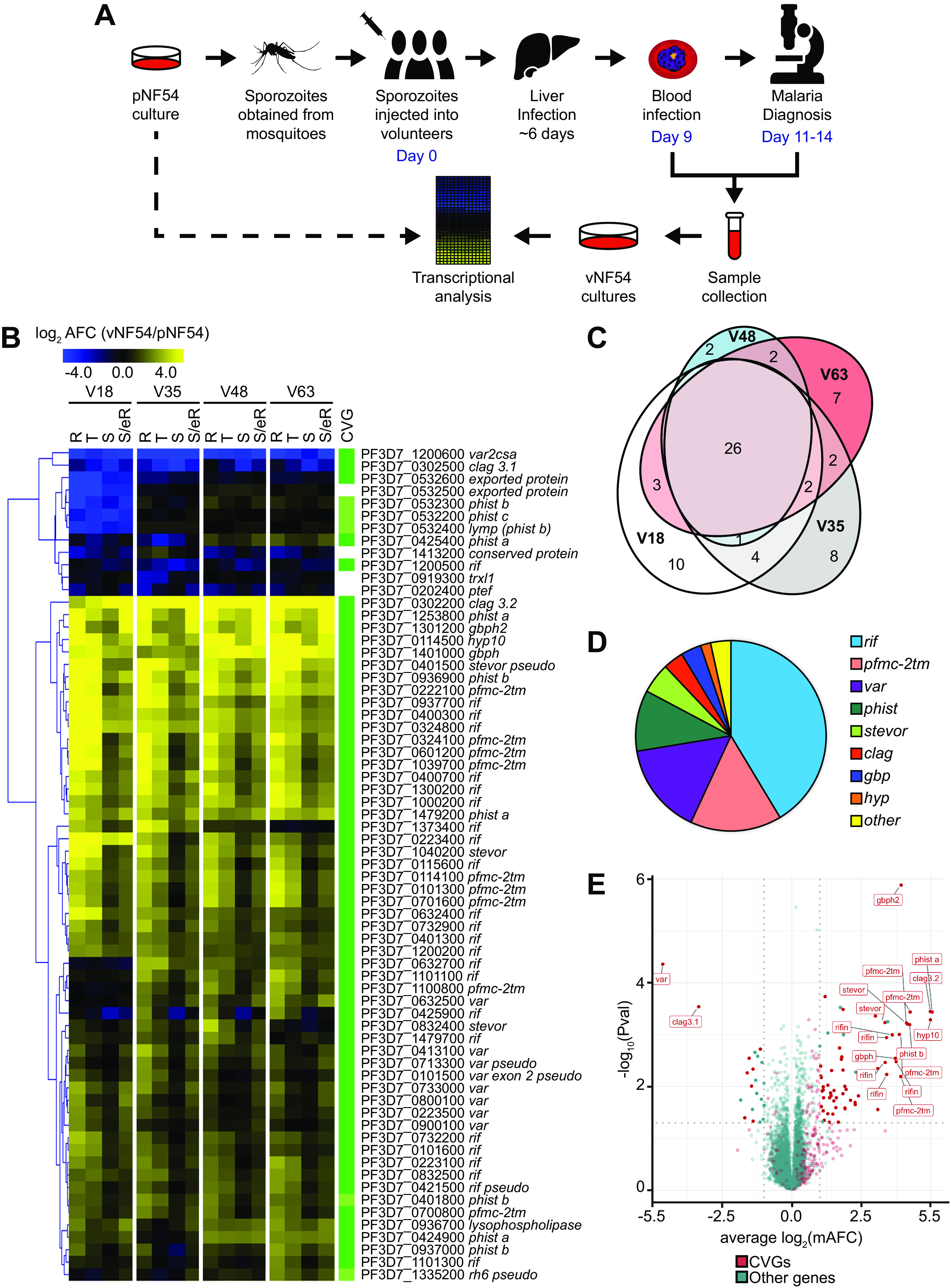
Genes differentially expressed between vNF54 and pNF54 lines. (A) Schematic of the study design comparing transcript levels between vNF54 lines obtained from volunteers participating in a controlled human malaria infection (CHMI) trial and the parental (premosquito) pNF54 line. (B) Heatmap of genes differentially expressed between vNF54 and pNF54 lines, ordered by hierarchical clustering. V18, V35, V48, and V63 are vNF54 lines obtained from different volunteers. Values are the log_2_ of the average fold change (AFC) of vNF54 versus pNF54 lines over four overlapping time intervals corresponding to the stages indicated (R, rings; T, trophozoites; S, schizonts; S/eR, schizonts and early rings). Only genes with an absolute value of the log_2_ of the maximum AFC (mAFC) of >2 (relative to pNF54) in at least one of the vNF54 lines are shown (see Materials and Methods for exclusion criteria). The column at the right indicates whether a gene was previously classified as a CVG (dark green; see Data Set S2) or belongs to a gene family in which other genes are CVGs (light green). Twelve genes had values out of the color scale range displayed. (C) Euler diagram showing the overlap between genes with an absolute value of the log_2_(mAFC) >2 (relative to pNF54) between the different volunteers. (D) Pie chart showing the distribution of genes with an absolute value of the log_2_(mAFC) of >2 between different gene families. (E) Volcano plot representing expression differences between vNF54 and pNF54 lines. Expression fold change values for each gene are the average of the log_2_(mAFC) in the four vNF54 lines. *P* values were calculated using an unpaired two-sided *t* test. CVGs are shown in red and other genes in green. Name labels are provided for genes with a mAFC of >10 and a *P* value of <0.01. Vertical dotted lines mark a log_2_(mAFC) of 1 or −1, whereas the horizontal dotted line marks a *P* value of 0.05.

We performed a time course genome-wide transcriptomic analysis across the full IDC of P. falciparum parasites obtained from a CHMI trial in which cryopreserved Sanaria NF54 sporozoites were injected into naive human volunteers (53). Parasites were cryopreserved on day 9 after infection and upon microscopy diagnosis on days 11 to 14. Transcriptomic analysis was performed using parasites collected from four different volunteers (V18, V35, V48, and V63 lines, here collectively termed vNF54) on the day of diagnosis. Parasites were thawed and cultured for the minimum number of cycles needed to obtain sufficient material (4 replication cycles) and then tightly synchronized (involving an additional cycle of replication) before harvesting RNA at defined time points of the IDC (10 to 15, 20 to 25, 30 to 35, and 40 to 45 h postinvasion [hpi]). In parallel, we obtained RNA at the same time points from two independent biological replicates of tightly synchronized cultures of the parental (premosquito) NF54 line (pNF54) (Fig. 1A). Transcript levels were determined using two-channel, long-oligonucleotide microarrays in which samples were hybridized against a common reference pool to obtain relative expression values (Cy5/Cy3). To quantify transcript-level differences between vNF54 and pNF54 lines, we calculated, for each gene, the maximum average fold change among overlapping time intervals of half the duration of the IDC (mAFC) (12) (see Materials and Methods).

10.1128/mBio.01636-21.7 DATA SET S2 List of P. falciparum clonally variant genes used for the analyses in this study. Download Data Set S2, XLS file, 0.7 MB. Copyright © 2021 Pickford et al. 2021 Pickford et al. https://creativecommons.org/licenses/by/4.0/ This content is distributed under the terms of the Creative Commons Attribution 4.0 International license.

There were 67 genes with a high-confidence mAFC of >4 (see Materials and Methods) in either direction [absolute value of the log_2_(mAFC) > 2] between at least 1 of the vNF54 lines and the pNF54 line, 21 of which had a mAFC of >16 (Fig. 1B and Data Set S1 in the supplemental material). The vast majority of differentially expressed genes had been previously identified as CVGs (Fig. 1B), either based on variant expression between isogenic lines (12) or because they carried the H3K9me3 or HP1 heterochromatin marks (54–57) (Data Set S2). Overall, the transcriptional changes (relative to pNF54) observed in the four vNF54 lines were highly similar (Fig. 1B), with the exception of a few genes showing a different pattern only in the V18 line. As a consequence, there was a large overlap in the genes differentially expressed (relative to pNF54) in each of the four vNF54 lines (Fig. 1C). Closer analysis revealed only a small number of genes with large transcript-level differences among the four vNF54 lines, and many of them showed a different pattern only in the V18 line, including a cluster of five downregulated neighbor genes in the distal subtelomeric region of chromosome 5 (Fig. S1).

10.1128/mBio.01636-21.1 FIG S1 Genes differentially expressed between parasites obtained from the different infected volunteers. Heatmap of the most differentially expressed genes between the four vNF54 lines (V18, V35, V48, and V63, obtained from four different volunteers), ordered by hierarchical clustering. Values are the log_2_ of the average fold change (AFC) of vNF54 versus pNF54 lines over four overlapping time intervals corresponding to the stages indicated (R, rings; T, trophozoites; S, schizonts; S/eR, schizonts and early rings). Only genes with an absolute value of the log_2_(mAFC) of >2 in at least one of the possible pairwise comparisons among the vNF54 lines (in both a direct comparison between vNF54 lines and using values normalized against the pNF54 line analyzed in parallel) are shown. See Materials and Methods for exclusion criteria. Four genes had values out of the range of the color scale displayed. The column at the right indicates CVGs according to the list in Data Set S2 (dark green) or genes that belong to a gene family in which other genes are CVGs (light green). Download FIG S1, PDF file, 0.2 MB. Copyright © 2021 Pickford et al. 2021 Pickford et al. https://creativecommons.org/licenses/by/4.0/ This content is distributed under the terms of the Creative Commons Attribution 4.0 International license.

10.1128/mBio.01636-21.6 DATA SET S1 Transcriptomic analysis of vNF54 and pNF54 lines. Download Data Set S1, XLS file, 0.2 MB. Copyright © 2021 Pickford et al. 2021 Pickford et al. https://creativecommons.org/licenses/by/4.0/ This content is distributed under the terms of the Creative Commons Attribution 4.0 International license.

The majority of genes differentially expressed in all vNF54 lines compared to pNF54 were expressed at higher levels in vNF54 lines, but a small number of genes were expressed at lower levels. The two most downregulated genes (mAFC > 10) were *var2csa* and *clag3.1*. The most upregulated genes in vNF54 lines (mAFC > 32) were a *hyp10* gene encoding an exported protein (PF3D7_0114500) (58), a *phist-a* gene (PF3D7_1253800), *clag3.2* (PF3D7_0302200), and a *pfmc-2tm* gene (PF3D7_0324100). Overall, the majority of genes that showed changes in expression between pNF54 and vNF54 lines belong to the large *pfmc-2tm*, *rif*, *var*, *stevor*, and *phist* CVG families encoding exported proteins (16–18, 58, 59) or to the smaller families *clag*, involved in solute transport (20), and glycophorin binding protein (*gbp*), encoding exported proteins of unknown function (58, 60, 61) (Fig. 1D). Given the overall similarity between the different vNF54 lines, we performed an additional analysis in which the different vNF54 lines were treated as replicates, which enables statistical analysis of the expression differences observed. The genes identified as most differentially expressed in this analysis were roughly the same as in the analysis of each vNF54 line separately, and *gpbh2* (PF3D7_1301200) was the most significantly upregulated gene in vNF54 lines (Fig. 1E and Data Set S1).

### Transcriptional changes in specific CVG families.

To analyze the expression patterns of CVGs within specific gene families, in addition to the transcript levels relative to a common reference pool (Cy5/Cy3 values), we also used the sample signal (Cy5 channel). The two-channel microarray approach used here is designed for hybridization against a common reference pool, which enables robust comparison of relative transcript levels between samples (3, 12, 62). However, the sample signal alone provides a semiquantitative estimate of the expression intensity of the genes, and it enables the identification of the predominantly expressed members of specific families (12), rather than informing only about the relative levels between samples.

**FIG 2:**
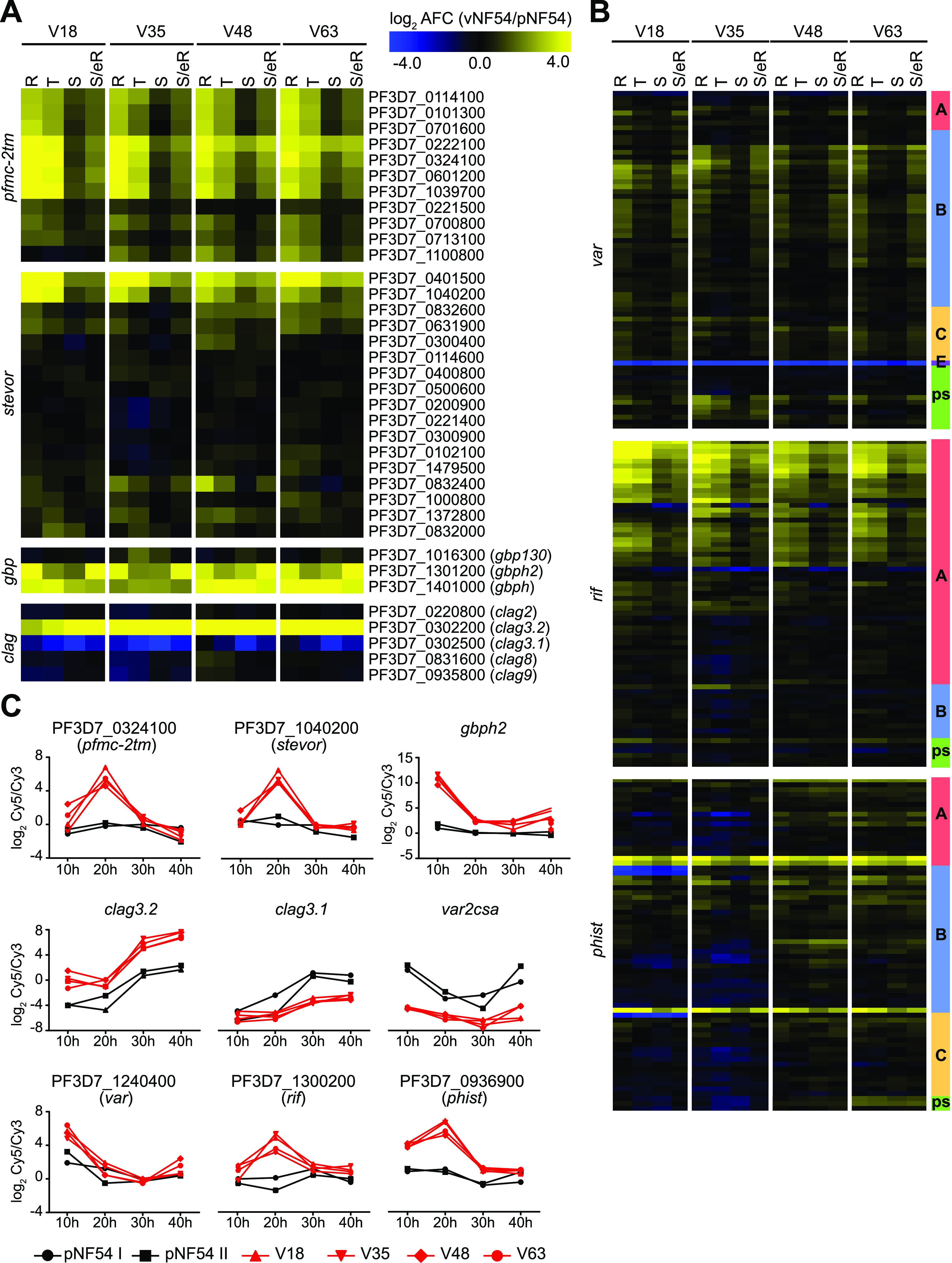
Transcriptional differences in specific CVG families. (A and B) Transcript-level changes in vNF54 lines relative to pNF54, as in Fig. 1B, for the CVG families indicated. See Materials and Methods for exclusion criteria. Eighteen genes (between the two panels) had values out of the color scale range displayed. In panel B, the subfamily of each gene (as annotated in PlasmoDB or in a previous study [12]) and pseudogenes (ps) are indicated in the column at the right. (C) Time course expression plots of selected genes of different CVG families. Values are the log_2_ of the sample versus reference pool ratio (Cy5/Cy3) in the vNF54 and pNF54 lines (*y* axis) and time after Percoll-sorbitol synchronization at which the sample was collected (*x* axis).

Analysis of the transcriptional differences between pNF54 and vNF54 lines in specific CVG families revealed different scenarios for different families (Fig. 2A and B and Fig. S2). Essentially all members of the *pfmc-2tm* family (39, 59, 63) were strongly upregulated in vNF54 lines (mAFC > 8 in all but three genes) (Fig. 2A and C), and this was confirmed by analyzing the Cy5 signal only, which revealed a large increase in the expression of the majority of *pfmc-2tm* genes (Fig. S2A). The analysis of a specific PfMC-2TM protein for which antibodies were available also revealed clearly increased abundance in a vNF54 line compared to pNF54 (Fig. S2B). In contrast, in the *var*, *rif*, *phist*, and *stevor* families, several specific genes were upregulated in vNF54 lines, but many other genes did not change, and, in some families, a few were downregulated. In the case of the mutually exclusively expressed *var* genes, vNF54 lines showed upregulation of many *var* genes, especially of type B, whereas the type E *var2csa* gene was strongly downregulated (Fig. 2B and C). Analysis of the Cy5 values revealed that *var2csa* was the predominantly expressed *var* gene in pNF54, whereas in vNF54 lines, multiple *var* genes were expressed at intermediate levels, similar to findings from previous CHMI studies (36, 38, 48, 52) (Fig. S2C). Considering that individual parasites express a single *var* gene, this result indicates that the parental population was relatively homogeneous, such that the vast majority of individual parasites expressed the same *var* gene (*var2csa*), whereas after passage through transmission stages the population became heterogeneous, with different individual parasites expressing different *var* genes. Some of the most highly expressed *var* genes in vNF54 lines coincided with the most highly expressed genes in a recent CHMI study using the 3D7 line to infect volunteers (52) (Fig. S3A), and preferential upregulation of type B *var* genes in parasites from the volunteers is also consistent with previous reports from other CHMI studies (36, 48, 52).

10.1128/mBio.01636-21.2 FIG S2 Expression intensity in the CVG families with the largest transcriptional differences between vNF54 and pNF54 lines. (A) Normalized Cy5 signal for *pfmc-2tm* genes in the four vNF54 lines (V18, V35, V48, and V63) and two replicates of the pNF54 line. (B) Western blot analysis of pNF54 and V63 schizonts with antibodies against a specific member of the PFMC-2TM family (PF3D7_ 0114100) and the loading control heat shock protein 70 (HSP70) in three independent biological replicates (I, II, and III). Bar chart shows the quantification of the relative PFMC-2TM band intensity (normalized by HSP70 band intensity). Data are presented as the average and SEM of three independent biological replicates. The difference between pNF54 and V63 was significant (*P < *0.05) using an unpaired two-sided *t*-test. (C to H) Same as panel A, but for genes of the *var* (C), *rif* (D), *stevor* (E), *phist* (F), *gbp* (G), and *clag* (H) families. Subfamilies are indicated for *var*, *rif*, and *phist* genes. In panels A, C, D, and H, expression values are at the time point of maximal expression of the gene family, i.e., 10 to 15 hpi for *var*, 20 to 25 hpi for *pfmc-2tm* and *rif*, and 30 to 35 hpi for *clag*. In panels E, F, and G, values are at the time of maximal expression for each line and gene because different genes of the *stevor*, *phist*, and *gbp* families have different times of maximal expression. Download FIG S2, PDF file, 0.3 MB. Copyright © 2021 Pickford et al. 2021 Pickford et al. https://creativecommons.org/licenses/by/4.0/ This content is distributed under the terms of the Creative Commons Attribution 4.0 International license.

10.1128/mBio.01636-21.3 FIG S3 Analysis of CVG expression patterns in another CHMI study. (A) Expression levels of *var* and *rif* genes in our study (Pickford) and in the Milne et al. study (52), ordered from highest to lowest expression in our data set. For our study, the median Cy5 signal of the vNF54 samples V18, V35, V48, and V63 at 10 to 15 hpi (*var*) or 20 to 25 hpi (*rif*) is shown. For the Milne study, the median of the Rlog expression value in volunteers (*n *= 12) infected with blood-stage parasites (Milne_Blood) and volunteers (*n *= 5) infected with sporozoites (Milne_Mosq.) is shown. Only genes for which data are available in the three datasets are shown. Eighteen genes had values out of the range of the color scale displayed. (B) CVG expression after different numbers of rounds of the IDC in the human circulation in the Milne et al. study. Box-and-whiskers plots showing the median Rlog values (boxes are quartiles, and whiskers are range) in the inoculum used to infect volunteers with blood-stage parasites (“Pre,” corresponding to a blood sample collected at day 13 from a volunteer infected by mosquito bite, i.e., after liver development and three rounds of the IDC; *n *= 2 technical replicates) and in volunteers infected with the inoculum (“Post,” corresponding to samples collected 7.5 to 10.5 days after infection by blood challenge with the inoculum; *n *= 12 volunteers). Genes with large differences between pNF54 and vNF54 lines and peak transcript levels at the ring stage (the only stage available in the Milne dataset) were included. (C) Pie chart showing the distribution between clonally variant genes (CVGs; specific families indicated) and nonclonally variant genes among the top 100 genes with the largest changes in median rlog values between “Pre” (inoculum) and “Post” samples in the Milne et al. study. Download FIG S3, PDF file, 0.3 MB. Copyright © 2021 Pickford et al. 2021 Pickford et al. https://creativecommons.org/licenses/by/4.0/ This content is distributed under the terms of the Creative Commons Attribution 4.0 International license.

In the *rif* family (18, 64–67), a large subset of genes encoding type A RIFINS were upregulated in all vNF54 lines, whereas no general differences were observed in genes encoding type B RIFINS. In the *stevor* and *phist* families (18, 58, 68), only a small subset of specific genes showed strong upregulation in vNF54 lines, essentially the same genes in all four lines (Fig. 2A to C). In the *rif*, *stevor*, and *phist* families, which participate in antigenic variation and show expression switches but do not show mutually exclusive expression (39, 67, 69), the analysis of the Cy5 signal revealed that, in spite of changes in the expression of some specific genes, the predominantly expressed genes are similar between pNF54 and vNF54 lines (Fig. S2D to F). None of the genes that showed increased transcript levels in vNF54 lines became the dominantly expressed gene in any of these families. Of note, the most highly expressed *rif* gene in our data set was the same as in a previous CHMI study (52) (Fig. S3A).

Two small CVG families, *gbp* (60) and *clag* (20), showed major changes in the expression of a large proportion of their genes. Two of the three members of the *gbp* family, *gbph* and *gbph2*, were among the most highly upregulated genes (mAFC > 16) in vNF54 lines (Fig. 2A and C and Fig. S2G). In all vNF54 lines, there was increased expression of *clag3.2* and reduced expression of *clag3.1*, consistent with our previous reverse transcriptase quantitative PCR (RT-qPCR) results showing that, in the pNF54 population, essentially all parasites express *clag3.1*, whereas in vNF54 lines, there is a mixture of some parasites expressing *clag3.1* and a majority of parasites expressing *clag3.2* (23). There was no major change in the expression of the other CVG of the *clag* family, *clag2* (32) (Fig. 2A and C and Fig. S2H).

The expression of other CVG families such as acyl-CoA synthetase (*acs*), acyl-CoA binding protein (*acbp*), lysophospholipase, exported protein kinase (*fikk*), *hyp1* to *hyp 17*, *surfin*, and families linked to erythrocyte invasion (*eba*, *pfrh*) was almost identical between pNF54 and vNF54 lines, with the exception of a *hyp10* (PF3D7_0114500) and a lysophospholipase gene (PF3D7_0936700) (Fig. S4A).

10.1128/mBio.01636-21.4 FIG S4 Transcriptional patterns in clonally variant gene families that show few differences between vNF54 and pNF54 lines. (A) Transcript level changes in vNF54 relative to pNF54 lines, as in Fig. 2, for the clonally variant gene families indicated. Genes are ordered according to hierarchical clustering. One gene had values out of the color scale range displayed. (B) Same as in panel A, but for well-established markers of sexual commitment or early gametocytes. Download FIG S4, PDF file, 0.2 MB. Copyright © 2021 Pickford et al. 2021 Pickford et al. https://creativecommons.org/licenses/by/4.0/ This content is distributed under the terms of the Creative Commons Attribution 4.0 International license.

### Most changes in CVG expression between vNF54 and pNF54 lines are determined at the epigenetic level.

The observation that the majority of genes differentially expressed between pNF54 and vNF54 lines are CVGs, which have been previously shown to be regulated by truly epigenetic mechanisms (25), strongly suggests that parasites recovered from the volunteers mainly differ from the parental line in their epigenetic makeup, rather than at the genetic level. To confirm this view, we sequenced the whole genome of the pNF54 and two vNF54 lines (V18 and V63) and found no genetic differences in either coding or noncoding regions that are likely to explain any of the transcriptional changes common to all vNF54 lines (Data Set S3). However, we observed one duplicated region and several large subtelomeric deletions in V18 that explain the reduced expression of several genes specifically in this parasite line, including the cluster of neighbor downregulated genes in the distal subtelomeric region of chromosome 5 (Data Set S3). Unexpectedly, in the V18 line, there were essentially no reads mapping to the deleted regions, indicating that these large deletions occur with a 100% prevalence. Parasites in the parental pNF54 line (as in any other isolate maintained in culture for a long time) are expected to be a mixture of individual parasites that have accumulated different mutations (70). Therefore, the presence of deletions with a 100% prevalence in the V18 line suggests that this line is genetically homogeneous because it originated from a single sporozoite infecting the liver. Consistent with this view, the majority of differential single nucleotide polymorphisms (SNPs) and small indels identified among the pNF54, V63, and V18 lines were unique to V18 and had a 100% prevalence in this line (Data Set S3). Of note, the volunteer from which the V18 line originated was part of a group of the CHMI trial that was inoculated under a scheme that resulted in blood infection in only one out of six volunteers (group 2, 2,500 P. falciparum sporozites [PfSPZ] in 50 μl, intramuscular injection), indicating that very few sporozoites were viable. In contrast, the other parasite lines analyzed (V35, V48, and V63) are from volunteers that received direct venous inoculation resulting in infection of 100% of the volunteers (53). Altogether, these results indicate that the V18 line is genetically homogeneous for the mutations because it likely originated from infection by a single sporozoite.

10.1128/mBio.01636-21.8 DATA SET S3 Full genome sequencing of vNF54 and pNF54 lines. Download Data Set S3, XLS file, 1.6 MB. Copyright © 2021 Pickford et al. 2021 Pickford et al. https://creativecommons.org/licenses/by/4.0/ This content is distributed under the terms of the Creative Commons Attribution 4.0 International license.

**FIG 3:**
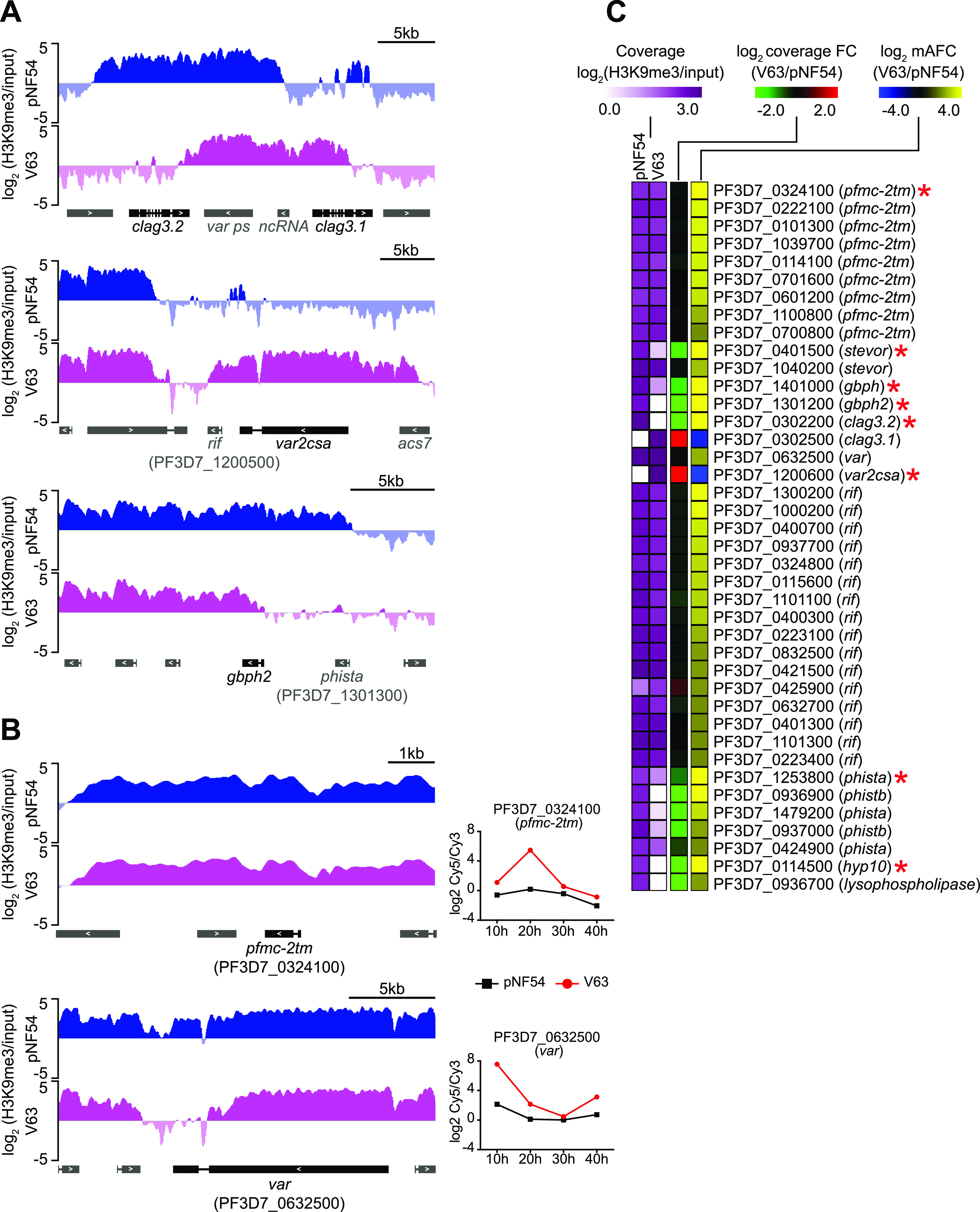
ChIP-seq analysis of vNF54 and pNF54 lines. (A and B) Distribution of normalized H3K9me3 signal relative to input in the pNF54 line and the vNF54 line V63. Representative genes are shown in which transcriptional changes between pNF54 and V63 are (*clag3.1*, *clag3.2*, *var2csa*, and *gbph2*) (A) or are not (*pfmc-2tm* and *var*) (B) accompanied by differences in heterochromatin distribution at their upstream regions. Genes are shown as rectangles (introns displayed as thin lines), with the direction of transcription indicated by arrowheads. The names of neighbor genes (in gray) with different levels of H3K9me3 between pNF54 and V63 are shown. The time course expression plot for the genes in panel B, not included in Fig. 2C, is shown. These two genes had the largest expression fold increase in V63 relative to pNF54 in the entire *pfmc-2tm* and *var* families. (C) H3K9me3 ChIP-seq coverage (from −1,000 bp or closest upstream gene to +500 bp from the ATG), coverage fold change (FC) between V63 and pNF54, and mAFC between V63 and pNF54 for genes showing an absolute value of the log_2_(mAFC) of >2 between V63 and pNF54. Genes with an absolute value of the log_2_(mAFC) >4 (values out of the color scale range displayed) are marked with an asterisk.

**FIG 4:**
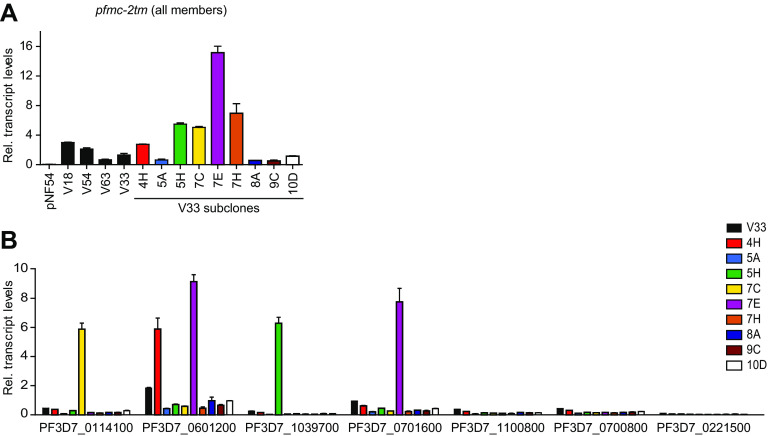
Expression of *pfmc-2tm* genes in subclones of a vNF54 line. (A) Transcript levels of *pfmc-2tm* genes (all members, analyzed with primers that amplify all the genes of the family) in pNF54, different vNF54 lines, and subclones of the V33 line at 20 to 25 h postinvasion (hpi). (B) Transcript levels of selected individual *pfmc-2tm* genes in V33 and V33 subclones. Transcript levels are normalized against serine-tRNA ligase (*serrs*). Data are presented as the average and SEM of two independent biological replicates.

Next, we performed comparative H3K9me3 chromatin immunoprecipitation followed by sequencing (ChIP-seq) analysis to determine the distribution of heterochromatin in one of the vNF54 lines (V63) and pNF54. In the genes showing the largest transcript-level differences between the two lines, higher expression was associated with reduced levels of heterochromatin in the upstream region and beginning of the coding sequence, whereas lower expression was associated with increased levels of heterochromatin (Fig. 3A and C and Data Set S4). This pattern was observed in 7 out of 8 genes with a mAFC of >16 between V63 and pNF54, and it occurred in genes from multiple families, including *stevor*, *gbp*, *clag*, *var*, *phist*, *hyp10*, and lysophospholipase. However, there were no apparent differences in heterochromatin prevalence between V63 and pNF54 at the putative regulatory regions of many differentially expressed genes from the large CVG families *pfmc-2tm*, *rif*, *var*, and *stevor* (Fig. 3B and C). The most plausible interpretation for this result is that individual genes of these families were activated in only small subsets of the parasites in V63, accounting for the increased transcript levels observed, but they remained silenced and in a heterochromatic state in the majority of parasites. Indeed, the analysis of a collection of subclones from the vNF54 line V33 (23), which also expressed *pfmc-2tm* genes at much higher levels than pNF54 (Fig. 4A), revealed that individual *pfmc-2tm* genes were silenced in the majority of subclones and expressed at very high levels in others (Fig. 4B). A similar scenario was observed for *rif* and *stevor* genes (Fig. S5). The expression patterns in recent subclones reflect the expression in individual parasites (12). Thus, these results indicate that individual *pfmc-2tm*, *rif*, and *stevor* genes were activated in only small subsets of parasites in V33 and also demonstrate population heterogeneity for the expression of these genes in a vNF54 line. For *var* genes, it has been previously established that in a population with a broad expression pattern, each individual gene is silenced in the majority of individual parasites (31, 36, 37). Together, these results indicate that, in the CVGs for which an association between increased transcript levels and lower levels of heterochromatin was not observed, this was explained by activation of the genes in only a small fraction of the parasites.

10.1128/mBio.01636-21.5 FIG S5 Expression of *stevor* and *rif* genes in subclones of a vNF54 line. Transcript levels of selected *stevor* (A) and *rif* (B) genes in pNF54, the V33 vNF54 line, and subclones of the V33 line at 20 to 25 h postinvasion (hpi). The two *stevor* genes included in the analysis had maximal expression at the trophozoite stage. Transcript levels are normalized against serine-tRNA ligase (*serrs*). Data are presented as the average and SEM of two independent biological replicates. Download FIG S5, PDF file, 0.1 MB. Copyright © 2021 Pickford et al. 2021 Pickford et al. https://creativecommons.org/licenses/by/4.0/ This content is distributed under the terms of the Creative Commons Attribution 4.0 International license.

10.1128/mBio.01636-21.9 DATA SET S4 ChIP-seq analysis of pNF54 and a vNF54 line. Download Data Set S4, XLS file, 1.2 MB. Copyright © 2021 Pickford et al. 2021 Pickford et al. https://creativecommons.org/licenses/by/4.0/ This content is distributed under the terms of the Creative Commons Attribution 4.0 International license.

### Changes in the expression of many CVGs are determined by a reset of epigenetic patterns during transmission stages.

For *var* and *clag3* genes, an epigenetic reset during transmission stages results in transcriptional heterogeneity in the parasite population at the onset of a blood infection (23, 36–38, 48, 50). To determine if the epigenetic memory for the transcriptional state of other CVGs is also lost during transmission stages, we compared the expression of *gbph2*, *pfmc-2tm*, and *stevor* genes between samples collected from the volunteers on day 9 or the day of microscopy diagnosis (for the samples used, day 11 or 14). Since liver stage development lasts ∼6 days (36, 48, 49, 71), day 9 samples correspond to parasites that multiplied in the human circulation for only one round of the IDC after egress from the liver (second-generation blood stages), whereas day 11 or 14 samples are from parasites that replicated for 1 or 3 additional cycles, respectively (third- or fifth-generation blood stages), according to previous estimations (49). We reasoned that if only within-host selection during the IDC was responsible for the differences between pNF54 and vNF54 lines and there was no epigenetic reset during transmission stages, day 9 samples would show expression levels intermediate between pNF54 and day 11 or 14 samples, as parasites with unfavorable expression patterns would be eliminated progressively. In contrast, if an epigenetic reset is the main determinant of the expression patterns observed, similar transcript levels would be expected between day 9 and day 11 or 14 samples (23).

**FIG 5:**
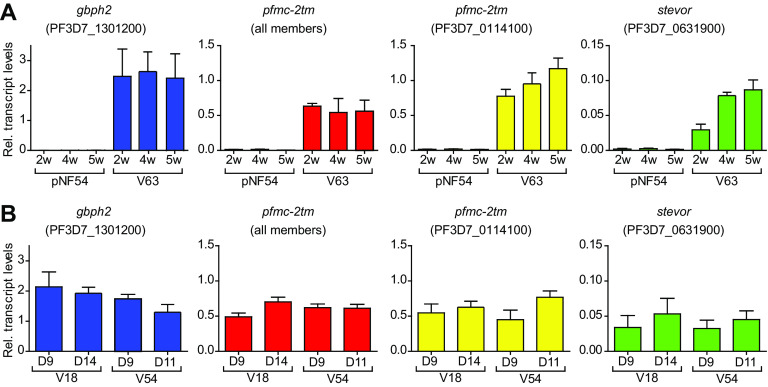
Changes in transcript levels associated with time in culture or time in the human circulation. (A) Effect of duration of growth under culture conditions on the expression of selected CVGs with different transcript levels between V63 (a vNF54 line) and pNF54. Relative transcript levels were determined by RT-qPCR after different times in culture, up to 5 weeks (w), in the pNF54 and V63 lines. RNA for transcriptional analysis was collected at the ring stage (10 to 15 h postinvasion [hpi]) for the *gbph2* gene and at the early trophozoite stage (20 to 25 hpi) for *pfmc-2tm* and *stevor* genes. For “*pfmc-2tm* (all members)”, primers that amplify all genes of the family were used. (B) Comparison of relative transcript levels between parasites collected at day 9 postinfection or at days 14 (V18) or 11 (V54) (day of microscopy diagnosis), determined as in the previous panel. Transcript levels are normalized against serine-tRNA ligase (*serrs*). Data are presented as the average and SEM of three independent biological replicates.

Because the parasitemia of day 9 samples was very low, obtaining sufficient material for RT-qPCR analysis required ∼7 cycles of *in vitro* growth, whereas only ∼4 cycles were needed for day 11 or 14 samples. Therefore, we first determined whether extended culture affected the expression levels of these genes. After 5 weeks in culture, transcript levels of *gbph2*, one *pfmc-2tm* gene, one *stevor* gene, and total *pfmc-2tm* family transcripts remained stable in both pNF54 and a vNF54 line (V63) (Fig. 5A). This result indicates that growth under *in vitro* conditions does not rapidly alter the expression of these genes and does not represent a confounding factor for the comparison of day 9 with day 11 or 14 samples. These experiments also confirmed the much higher transcript levels for these genes in vNF54 than in pNF54, and they revealed that they are virtually silenced in the latter.

Next, we compared the transcript levels of these genes between parasites collected on day 9 or the day of microscopy diagnosis in two vNF54 lines (V18 and V54, with microscopy diagnosis on days 14 and 11, respectively), which revealed no large differences (Fig. 5B). These results indicate that multiplication for a small number of rounds of the IDC in the blood of a naive human host does not have a major impact on the expression of *gbph2*, *pfmc-2tm*, and *stevor* genes. This is consistent with the idea that their increased expression in vNF54 lines compared to pNF54 was established before parasites reached the blood circulation, i.e., during transmission stages, as a consequence of an epigenetic reset. The results from a recent CHMI study in which blood-stage parasites from a volunteer infected via mosquito bite were blood passaged to new volunteers also support this idea (52, 72). Transcriptional comparison of parasites collected before and after blood growth in the new volunteers (three to five cycles), analogous to our day 9 versus day 11 or 14 comparison, revealed no difference in the expression of *var* or *rif* genes (52). We analyzed, in that data set, the expression of other CVGs and also observed no consistent changes (Fig. S3B). The majority of differences between samples collected before and after blood growth in the new volunteers occurred in genes that are not CVGs (Fig. S3C).

### Phenotypic comparison of pNF54 and vNF54 lines.

**FIG 6:**
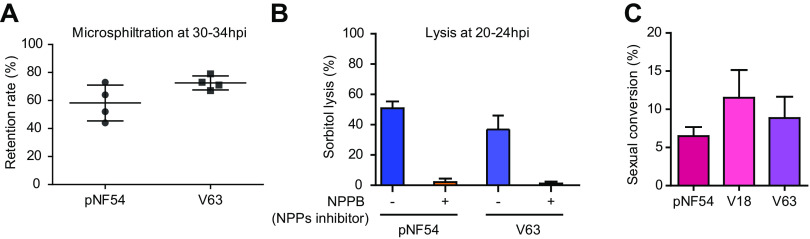
Phenotypic comparison of vNF54 and pNF54 lines. (A) Retention in microsphiltration assays of pNF54 and a vNF54 line (V63). Data are presented as the average, SEM, and individual data points of four independent biological replicates. (B) Sorbitol lysis assays for pNF54 and V63 in the absence or presence of NPPB (an inhibitor of new permeation pathways [NPPs]). Data are presented as the average and SEM of three independent biological replicates. (C) Sexual conversion rate (proportion of parasites that convert into sexual forms) of pNF54 and two vNF54 lines (V18 and V63). Data are presented as the average and SEM of three independent biological replicates. No significant difference (*P < *0.05) was observed between pNF54 and vNF54 lines in any of the panels, using an unpaired two-sided *t* test.

We performed exploratory experiments to determine if the transcriptional differences observed between pNF54 and vNF54 lines result in measurable functional differences. We focused on phenotypes that are mainly determined by CVGs and for which assays were available in our laboratories. Since many of the genes showing differential expression are exported to the erythrocyte cytoplasm or membrane (18, 58, 59) and some directly impact the mechanical properties of infected erythrocytes (i.e., *stevor*) (73), we compared the membrane deformability at the trophozoite stage of pNF54- and vNF54 (V63)-infected erythrocytes using a microsphiltration assay. While V63 showed a higher retention rate, indicative of lower deformability, the difference was not significant (Fig. 6A).

Since we observed changes in the expression of *clag3* genes, which determine solute uptake in infected erythrocytes (20), we compared sorbitol permeability between pNF54 and vNF54 lines. Sorbitol uptake resulting in red cell lysis was observed at similar levels in both lines (Fig. 6B) as expected, given that only the simultaneous silencing of the two *clag3* genes, which did not occur in either pNF54 or vNF54, is known to prevent sorbitol uptake (22). Lastly, we compared the sexual conversion rates of pNF54 and vNF54 lines (V18 and V63), which revealed no significant differences (Fig. 6C). This is consistent with the similar transcript levels of *pfap2-g*, the master regulator of sexual conversion (74), and of early gametocyte markers between pNF54 and vNF54 lines (Fig. S4B).

## DISCUSSION

Here, we provide the first genome-wide transcriptional characterization across the full IDC of P. falciparum parasites obtained during the initial days of a blood infection in malaria-naive humans and compare it with the transcriptome of parasites with the same genome but maintained under *in vitro* culture conditions. We also compared the genome-wide distribution of heterochromatin between parasites obtained from the infected humans or in culture. We found that the largest expression differences occur in CVGs and are associated with epigenetic changes in the distribution of heterochromatin, and we provide an accurate view of how parasites use their CVGs when they establish a new blood infection after egress from the liver. We also show that passage through transmission stages results in a reset of the epigenetic memory involving multiple CVG families.

The majority of changes between premosquito parasites and parasites obtained from the infected volunteers occurred in gene families involved in processes such as antigenic variation, erythrocyte remodeling, or solute transport, whereas other CVG families showed few alterations. Even within the same family, subgroups of genes with different predicted functions showed different expression patterns: type A RIFINS, predicted to encode proteins localized at the infected erythrocyte surface and involved in rosetting (18, 67), were generally upregulated in parasites collected from the volunteers, but not type B RIFINS with different predicted function and localization. The most dramatic changes were observed in the *pfmc-2tm*, *gbp*, *var*, and *clag* families, whereas other families, such as *rif*, *stevor*, or *phist*, showed important transcript-level differences for several specific genes, but the predominantly expressed genes and global expression levels of the family were not altered. Together, these observations suggest that some CVG families have high plasticity in their expression patterns, whereas others have clearly “preferred” patterns.

While the epigenetic state of CVGs is transmitted from one generation of asexual parasites to the next (11, 17, 25), it has been proposed that an “epigenetic reset” may occur during transmission stages such that the epigenetic memory for the transcriptional state of all CVGs is erased and new patterns of expressed and silenced CVGs are established stochastically (75). In the murine malaria parasite Plasmodium chabaudi, mosquito-transmitted parasites were less virulent than those transmitted by blood passage and expressed a broader repertoire of CVGs (75, 76). This led some authors to postulate that repeated cycles of the IDC progressively select for parasites with CVG expression patterns associated with increased fitness, and passage through transmission stages results in a reset of the epigenetic patterns and lower virulence (75), although evidence for this model in human malaria is lacking. In P. falciparum, an epigenetic reset during transmission stages was previously demonstrated for *var* and *clag3* genes (23, 36–38). Here, we observed clearly distinct expression patterns between premosquito parasites and parasites obtained from the volunteers for genes from multiple additional CVG families (*gbp*, *pfmc-2tm*, *stevor*, *rif*, *phist*, lysophospholipase, and *hyp10*) and similar expression patterns between day 9 and day 11 or 14 samples in all the CVG families analyzed (*gbp*, *pfmc-2tm*, and *stevor*). Similarly, previous analyses of parasites from CHMI trials showed no consistent CVG expression changes associated with an increasing number of multiplication cycles in the blood of naive humans (36, 52). These results are consistent with a general epigenetic reset of CVGs during transmission stages, although it is also possible that in CVGs for which no transcriptional changes were observed between pNF54 and vNF54 lines, the epigenetic memory is maintained throughout all transmission stages.

We postulate that during transmission stages, the epigenetic memory for multiple CVG families is erased, and later on, new epigenetic patterns are established stochastically such that they differ among individual parasites. However, while the state of a gene in an individual parasite is established stochastically, the probability of the gene acquiring an active or a silenced state may be hardwired and dictated by the underlying DNA sequence. This probabilistic scenario could explain the similar CVG expression patterns observed among the different volunteers and also the relatively constant patterns observed in some gene families. For some CVGs, the active or the silenced state may be strongly favored. Given that in the V18 line, which appears to have originated from a single sporozoite invading the liver, we observed CVG expression patterns similar to the other vNF54 lines, including a broad expression pattern of *var* genes indicative of transcriptional heterogeneity, we postulate that new epigenetic patterns are established during liver development. The stage at which the previous epigenetic patterns are erased is not known, but given the profound heterochromatin remodeling already observed in gametocytes and mosquito stages, it is likely to occur before parasites reach the liver (55, 77, 78).

Of note, in many genes from large multigene families, we observed large transcript-level fold differences that were not accompanied by measurable changes in the levels of H3K9me3 occupancy. We found that in the parasite populations obtained from the volunteers, individual genes of these families were active in only a small subset of the parasites, which still could result in a large fold increase in transcript levels if fewer parasites expressed it in the premosquito population (e.g., a 10-fold increase if a gene was active in 10% of the parasites collected from the volunteers but only in 1% of the premosquito population). This would result in a minor difference in the heterochromatin levels (e.g., 90% versus 99% of the parasites would have the gene from the example above in a heterochromatic state), which is not detectable in a comparative ChIP-seq analysis. Therefore, our results do not indicate that activation of many *var*, *rif*, or *pfmc-2tm* genes occurred independently of heterochromatin changes but, rather, reflect that activation occurred in only a small fraction of the parasites in the population, and reductions in heterochromatin levels were undetectable. Indeed, in the parental line, the *var2csa* gene was in an active state in essentially all the parasites, and this was clearly associated with the absence of heterochromatin at this locus, consistent with previous reports for the epigenetic regulation of *var* genes (28, 30).

Altogether, a model emerges in which a blood infection in a new host starts with a transcriptionally heterogeneous parasite population, consisting of a mixture of individual parasites with different combinations of active and silenced CVGs from multiple gene families. This constitutes the basis of a bet-hedging adaptive strategy because transcriptional heterogeneity results in phenotypic and antigenic diversity that increases the chances of survival of the parasite population in the unpredictable conditions of the blood of a new human host. Our data provide an accurate picture of how parasites use their CVGs in this initial phase of a blood infection, revealing that a higher proportion of individual parasites (compared to culture conditions) have in an active state the majority of genes of the *pfmc-2tm* and *gbp* families and specific genes of the *rif* (type A), *phist*, *stevor*, and a few other CVG families. Additionally, we demonstrate transcriptional heterogeneity for the expression of *rif*, *stevor*, and *pfmc-2tm* genes in addition to *var* and *clag3* genes (23, 36, 38, 48, 52). While in our study we could not characterize the long-term expression patterns of CVGs because infections were terminated as soon as parasites were detected by light microscopy or symptoms appeared, current knowledge suggests that during chronic blood infection, the expression of CVGs is governed by low-frequency switches and dynamic natural selection of parasites with expression patterns that confer high fitness as the conditions of the host fluctuate (9–11, 50). Future research will be needed to fully characterize the precise phenotypic and antigenic differences that result from specific changes in CVG expression.

## MATERIALS AND METHODS

### Human samples and parasite culture.

The P. falciparum NF54 line at Sanaria (pNF54) and lines derived from human volunteers participating in a CHMI study with Sanaria NF54 sporozoites (vNF54 lines) (53) were cultured in B+ erythrocytes at 3% hematocrit in RPMI 1640-based culture medium supplemented with 10% human serum under continuous shaking at 100 rpm and in a 5% CO_2_, 3% O_2_, and 92% N_2_ atmosphere.

### Transcriptomic analysis using microarrays.

Time course transcriptomic analysis was performed using cultures tightly synchronized to a 5-h age window, which was achieved by Percoll purification of schizonts followed by sorbitol lysis 5 h later (79). Sixty-milliliter cultures at 3% hematocrit and ∼10% parasitemia were split into four flasks that were cultured undisturbed for different periods of time (10, 20, 30, or 40 h) before harvesting in TRIzol and freezing at −80°C. Rather than splitting into identical cultures, larger volumes (3- to 4-fold) were used for the early time points because young (ring-stage) parasites contain a smaller amount of RNA per parasite. Cultures for RNA collection at late stages were diluted to ∼5% parasitemia with fresh erythrocytes (just after splitting) to prevent culture collapse.

RNA was purified using the TRIzol method, and cDNA was synthesized by reverse transcription (starting with 5 to 10 μg of RNA), purified, and labeled as previously described (62). Samples were analyzed using two-color long oligonucleotide-based custom Agilent microarrays. The microarray design was based on Agilent design AMADID 037237 (62), modified by adding new probes for genes lacking unique probes and for some ncRNAs and reporter genes (new designs, AMADID 084561 and AMADID 085763) and reannotated according to a BLAST analysis (80). All samples (200 to 500 ng), labeled with Cy5, were hybridized against an equal amount of a common reference pool labeled with Cy3, consisting of a mixture of equal amounts of cDNA from rings, trophozoites, and schizonts of pNF54. Microarray hybridization was performed as previously described (62). Images were acquired using a microarray scanner (G2505C; Agilent Technologies) located in a low-ozone hood.

### Microarray data analysis.

Initial processing of microarray data, including linear and locally weighted scatterplot smoothing (LOWESS) normalization, was performed using the Feature Extraction software (Agilent) with default options. The next steps of the analysis were performed using Bioconductor in an R environment (R version 3.5.3). For each individual sample and channel (Cy3 and Cy5), background signal was calculated as the median of the 100 lowest signal probes. Probes with both Cy3 and Cy5 signals below three times the array background in all samples were excluded from further analysis. Gene-level log_2_(Cy5/Cy3) values and statistical estimation of parasite age (81) were computed as previously described (12). For each gene, the log_2_(Cy5/Cy3) values were plotted against the statistically estimated culture age (in hpi), and the plots were divided into four overlapping time intervals of identical length that roughly corresponded to the ring, trophozoite, schizont, and late-schizont/early-ring stages (12). For each gene and time interval, the average expression fold change (AFC) between each vNF54 and its control pNF54 line (the replicate of pNF54 analyzed in parallel) was calculated from the difference in the area under the curve in the log_2_(Cy5/Cy3) versus estimated age plots. The maximum AFC (mAFC) was the value of the AFC in the time interval at which it had the highest absolute value. All tRNAs were excluded from further analysis because our method is not suitable for the analysis of tRNA expression (it tends to show large technical variability). For the identification of genes differentially expressed among pNF54 and vNF54 lines (i.e., Fig. 1B to D, Fig. 2, Fig. S1 in the supplemental material, and Data Set S1), genes with expression intensity (Cy5 channel) values that were in all samples within the lowest 20th percentile were excluded because expression differences in genes expressed at near-background levels are of low confidence. Based on this criterion, we excluded 17 out of 103 genes with an absolute value of the log_2_ mAFC (vNF54/pNF54) of >2 (Fig. 1B to D), 131 out of 889 genes with a value of >1 (Data Set S1), and 4 out of 42 genes with differential expression among vNF54 lines (Fig. S1; see below). Additionally, we excluded transcripts that lack a PlasmoDB ID and genes with apparent artifacts according to visual inspection (i.e., genes with large expression differences observed only at time intervals that do not correspond to their peak expression) from the list of top differentially expressed genes presented in Fig. 1B to D (6 and 13 genes, respectively) and Fig. S1 (7 and 4 genes, respectively). For the analysis presented in Fig. S1, we compared transcript levels directly between the different vNF54 lines. Since differences observed between lines that were not analyzed in parallel may potentially be attributable to a batch effect, we only included genes with an absolute value of the log_2_ mAFC of >2 (in any of the possible pairwise comparisons) among the vNF54 lines compared both directly and after normalizing against the pNF54 line analyzed in parallel. Heatmaps and hierarchical clustering based on Euclidean distance were generated using TMEV 4.9 (82). For the generation of volcano plots, samples from the different volunteers were treated as replicates, and an unpaired two-sided *t* test was performed between volunteer and parental NF54 replicates.

### RT-qPCR transcriptional analysis.

RNA was purified from parasite samples collected in TRIzol (Invitrogen) using the RNeasy minikit (Qiagen) as previously described (23, 79). Next, purified RNA was reverse transcribed using the reverse transcription system (Promega) alongside parallel reactions without reverse transcriptase to exclude gDNA contamination. Quantitative PCR to analyze cDNAs was performed in triplicate wells using the Power SYBR green master mix (Applied Biosystems) in a StepOnePlus real-time PCR system, essentially as previously described (26, 79). Relative transcript levels were calculated using the standard curve method and the normalizing gene serine-tRNA ligase (*serrs*), which shows stable transcript levels across the IDC. The primers used for qPCR are described in Data Set S5.

10.1128/mBio.01636-21.10 DATA SET S5 Oligonucleotides used in this study. Download Data Set S5, XLS file, 0.03 MB. Copyright © 2021 Pickford et al. 2021 Pickford et al. https://creativecommons.org/licenses/by/4.0/ This content is distributed under the terms of the Creative Commons Attribution 4.0 International license.

### Whole-genome sequencing.

PCR-free whole-genome Illumina sequencing was used to sequence the whole genome of the parental NF54 line alongside two vNF54 lines (V18 and V63). Genomic DNA was sheared to ∼150 to 400 bp using a Covaris S220 ultrasonicator, and the NEBNext Ultra DNA library prep kit for Illumina was used for library preparation with specific paired-end TruSeq Illumina adaptors for each sample. Due to the high AT content of the P. falciparum genome, the end repair incubation step at 65°C was omitted. After quality control using the Bioanalyzer DNA high sensitivity kit (Agilent) and quantification using the Kapa library quantification kit (Roche), libraries were sequenced using the Illumina HiSeq2500 system. Over 6 million 125-bp paired-end reads were obtained for each sample.

For the data analysis, read quality was checked (FastQC program), and adaptors were trimmed (Cutadapt program) before mapping sequenced reads to the PlasmodDB P. falciparum 3D7 reference genome version 46 (https://plasmodb.org/plasmo/) using the BWA-MEM alignment algorithm. Next, GATK UnifiedGenotyper was used to perform variant calling based on GATK best practices to identify SNPs and small indels. GATK Variant Filtration was used to filter out variants with low calling quality (Phred QUAL < 20) or low read depth (DP < 20). Differences in SNP/indel allelic frequency between the different strains were calculated for each SNP/indel, and those showing a <50% difference were filtered out. GenomeBrowse (Golden Helix) was used to visualize alignments and variants, as well as for the detection of large subtelomeric deletions or duplications.

### ChIP-seq experiments and analysis.

ChIP-seq experiments were performed essentially as previously described (80). In brief, chromatin was extracted from saponin-lysed synchronous parasite cultures at the late-trophozoite/early-schizont stage using the MAGnify chromatin immunoprecipitation system (Life Technologies) (34). After cross-linking and washing, chromatin was sonicated with an M220 sonicator (Covaris) at 10% duty factor, 200 cycles per burst, and 140 W of peak incident power for 10 min. Next, 4 μg of chromatin were immunoprecipitated overnight at 4°C with 8 μg of antibody against H3K9me3 (Diagenode; catalog no. C15410193) previously coupled to protein A/G magnetic beads provided in the kit. Samples were then washed, de-cross-linked, and eluted following the MAGnify ChIP system recommendations but, at all times, avoiding high temperatures that could result in the denaturation of extremely AT-rich intergenic regions. De-cross-linking, proteinase K treatment, and elution were performed at 45°C (for 2 h, overnight, and 1.5 h, respectively).

Using a protocol adapted to a genome with an extremely high AT richness (83), libraries for Illumina sequencing were prepared from 5 ng of immunoprecipitated DNA. After end repair and addition of 3′ A overhangs, NEBNext multiplex oligos for Illumina (NEB; catalog nos. E7335 and E7500) were ligated. Agencourt AMPure XP beads (Beckman Coulter) were used for the purification steps, and the libraries were amplified (9 amplification cycles) with the Kapa HiFi PCR kit (Kapa Biosystems) in Kapa HiFi fidelity buffer (5×). Finally, 0.9× AMPure XP beads were used to purify the amplified libraries and remove adapter dimers. After library size analysis using a 4200 TapeStation system (Agilent Technologies) and quantification using the Kapa library quantification kit (Roche), sequencing was performed with a HiSeq2500 system (Illumina), obtaining 6 to 10 million 125-bp paired-end reads per sample.

Reads were mapped to the 3D7 reference genome using the Bowtie2 local alignment algorithm. Differential peak calling was performed using MACS2 (v2.2.7.1) following the author’s recommendations. A first round of peak calling for every sample was performed using the MACS2 callpeak command with parameters as follows: -f BAMPE -g 2.41e7 --fe-cutoff 1.5 --nomodel --extsize 150, and then differential peaks were called using the bdgdiff command with parameters as follows: -g 250 -l 300 --cutoff 5. Results were annotated using custom scripts in Python against the P. falciparum 3D7 annotation in PlasmoDB (v46). The Integrative Genomics Viewer (IGV) was used to visualize the data.

H3K9me3 coverage across the genome for each sample was calculated using the DeepTools (v.3.5.0) BamCompare command. After normalizing to reads per kilobase per million (RPKMs), coverage was defined as the log_2_(IP/input) and computed for 100-bp intervals. For each gene, we calculated the average coverage for the 1,000 bp upstream plus the first 500 bp of the coding sequence because this is the region in which heterochromatin is typically associated with a silenced state.

### Determination of sexual conversion rates.

Sexual conversion rates were measured by treating sorbitol synchronized ring-stage cultures with 50 mM *N-*acetyl-d-glucosamine (GlcNAc) (Sigma-Aldrich; catalog no. A32869) for 5 days to eliminate asexual parasites. The sexual conversion rate was calculated as the gametocytemia at day 5 relative to the initial rings parasitemia at day 0 (the day of adding GlcNAc), as previously described (84). Initial parasitemia was measured by flow cytometry (85) and gametocytemia by light microscopy quantification of Giemsa-stained smears.

### Microsphiltration assay.

In order to compare the deformability of erythrocytes infected with pNF54 and vNF54 (V63), we used a microsphiltration assay (86). Calibrated metal microspheres (96.50% tin, 3.00% silver, and 0.50% copper; Industrie des Poudres Sphériques) with two different size distributions (5 to 15 μm and 15 to 25 μm in diameter) were used to create a matrix within an inverted 1,000-μl antiaerosol pipette tip (Neptune). For this, 4 g of dry microspheres of each size range were resuspended in 12 ml of parasite culture medium (with 10% human serum), and 400 μl of this microsphere suspension was added into the tip and left to settle and form a 3- to 4-mm-thick layer above the tip filter. Next, 600 μl of tightly synchronized 30- to 34-hpi cultures (1 to 6% parasitemia) were placed on top of the microsphere layer and then perfused through the microsphere matrix at a flow rate of 60 ml/h using an electric pump (Syramed SP6000; Arcomed Ag), followed by a wash with 5 ml of culture medium.

Samples collected after perfusion through the matrix (in triplicate) and the original culture (not passed through the matrix) were analyzed by flow cytometry. For this, 2 μl of erythrocytes pellet were collected in 200 μl of RPMI and washed twice with 200 μl of phosphate-buffered saline (PBS), incubated for 25 min at room temperature with SYBR green (1:2,000 dilution of Sigma S9430 solution), washed twice again with 200 μl of PBS, and finally resuspended in 1.5 ml of PBS. The parasitemia of each sample was then measured with a BD Accuri C6 cytometer.

### Sorbitol lysis assay.

To test sorbitol sensitivity, tightly synchronized 20- to 24-hpi pNF54 and vNF54 (V63) cultures were treated with a sorbitol-containing isosmotic solution (300 mM sorbitol supplemented with 10 mM HEPES, 5 mM glucose, and adjusted to pH 7.4) or the same isosmotic solution with 100 μM of the general anion channel inhibitor 5-nitro-2-(3-phenylpropylamino) benzoic acid (NPPB, Sigma-Aldrich), which inhibits malarial new permeation pathways (87). Parasitemia was determined before (time zero) and after (time 60) incubation at 37°C for 1 h, using flow cytometry. The lysis percentage was calculated for each condition using the following formula: % lysis = [1 − (parasitemia time 60 min/parasitemia time zero)] × 100.

### Western blotting.

Synchronous cultures containing mainly ∼40-hpi schizonts were purified by magnetic isolation, divided into pellets of approximately 2.5 × 10^6^ schizonts, and stored frozen at −80°C. After thawing, proteins were denaturized in SDS-PAGE protein loading buffer for 5 min at 95°C and resolved by SDS-PAGE on 4 to 12% bis-Tris Criterion XT precast gels (Bio-Rad), transferred to a polyvinylidene difluoride (PVDF) membrane, and blocked for at least 1 h in 1% casein blocking buffer (Sigma). Membranes were incubated overnight at 4°C with the following primary antibodies: purified mouse antiserum against a PfMC-2TM protein (PF3D7_0114100) at 1:200 (kindly provided by Catherine Braun-Breton) (88) and mouse anti-HSP70 antibody (89) at 1:2,000. Membranes were then incubated for 1 h at room temperature with horseradish peroxidase-conjugated anti-mouse IgG secondary antibody (Promega) at 1:10,000, and peroxidase was detected using the Pierce chemiluminescence system (Pierce) following the manufacturer’s instructions. To control for equal loading, parts of the membranes corresponding to different molecular weight ranges were separately hybridized with different antibodies. Signal quantification was performed using ImageJ software.

### Data availability.

The microarray and ChIP-seq data have been deposited in the GEO database with accession numbers GSE166258 and GSE166390, respectively. ChIP-seq data can be visualized at the UCSC genome browser using the following link: http://genome.ucsc.edu/s/apickford/apickford_lmichel. Whole-genome sequence data have been deposited at the Sequence Read Archive (SRA) database with accession number PRJNA699845. The scripts used for microarray data analysis are available in GitHub (https://github.com/CortesMalariaLab/A_Pickford_CHMItranscriptomic).
