# Supplementary figures and images for: Expression Patterns of Plasmodium falciparum Clonally Variant Genes at the Onset of a Blood Infection in Malaria-Naive Humans
Source: mBio. 2021 Aug 3;12(4):e01636-21. doi: 10.1128/mBio.01636-21 (PMC8406225; doi:10.1128/mBio.01636-21)

$\log_2$  AFC (vNF54/pNF54)

-4.0 0.0 4.0

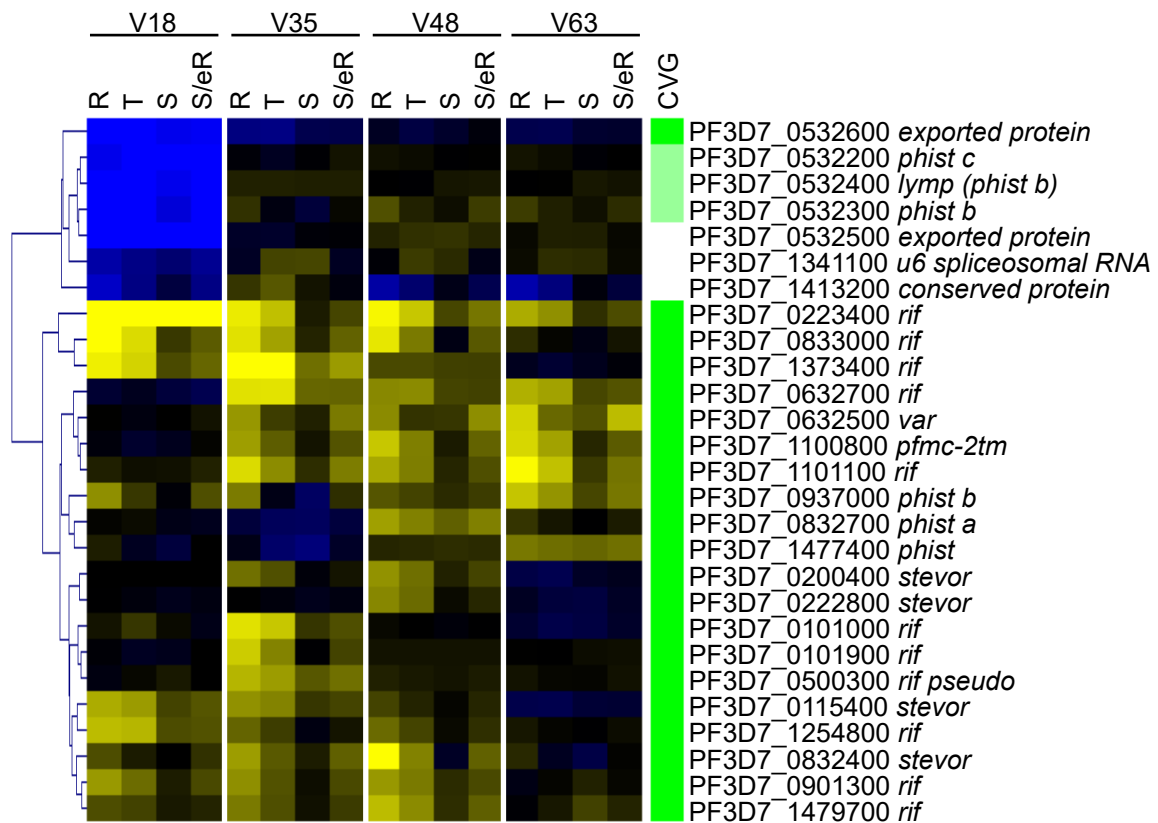

Supplement: FIG S1 [file mbio.01636-21-sf001.pdf]

**A**

*pfmc-2tm*

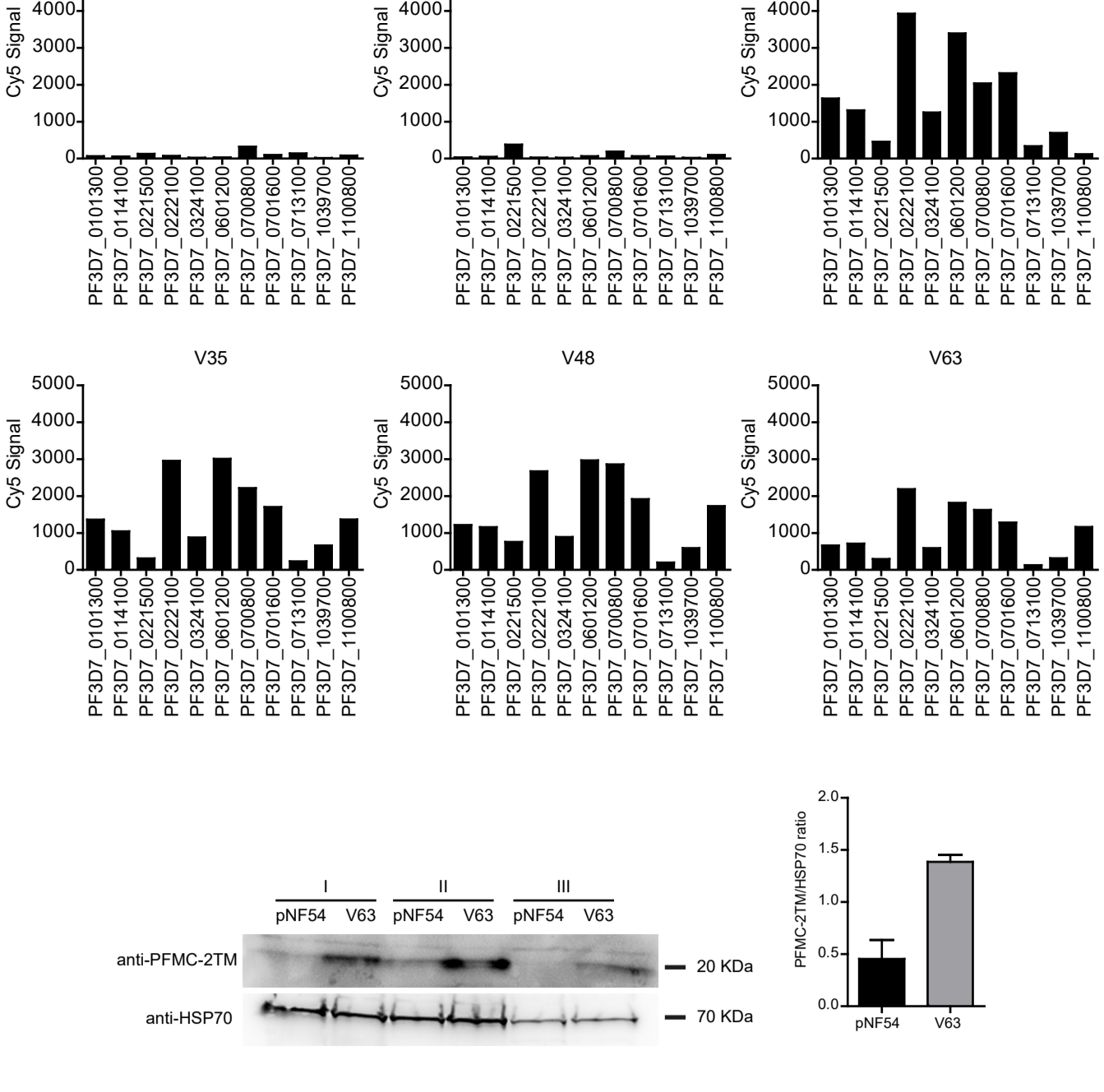

**B**

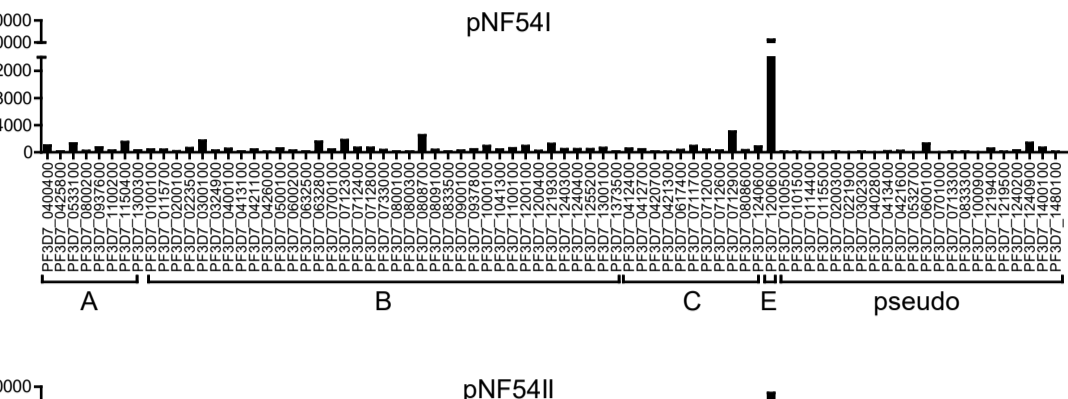

**C**

*var*

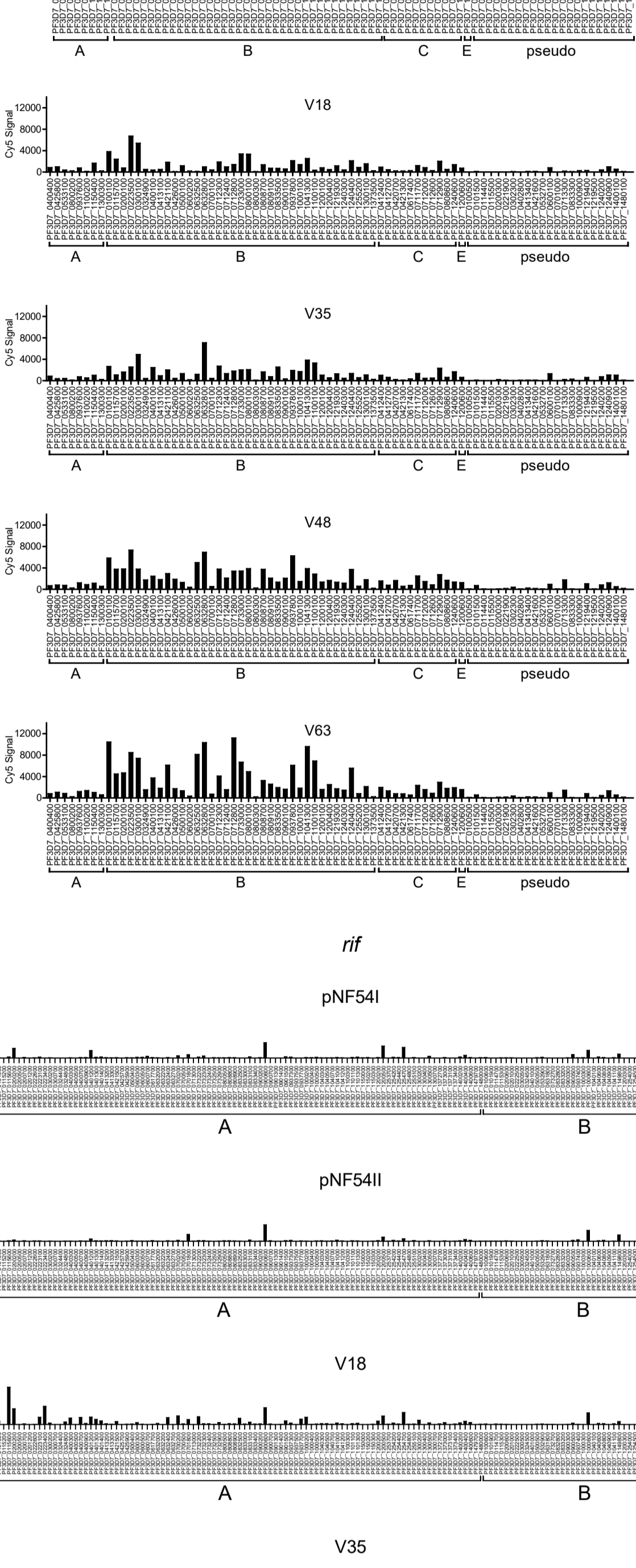

**D**

*rif*

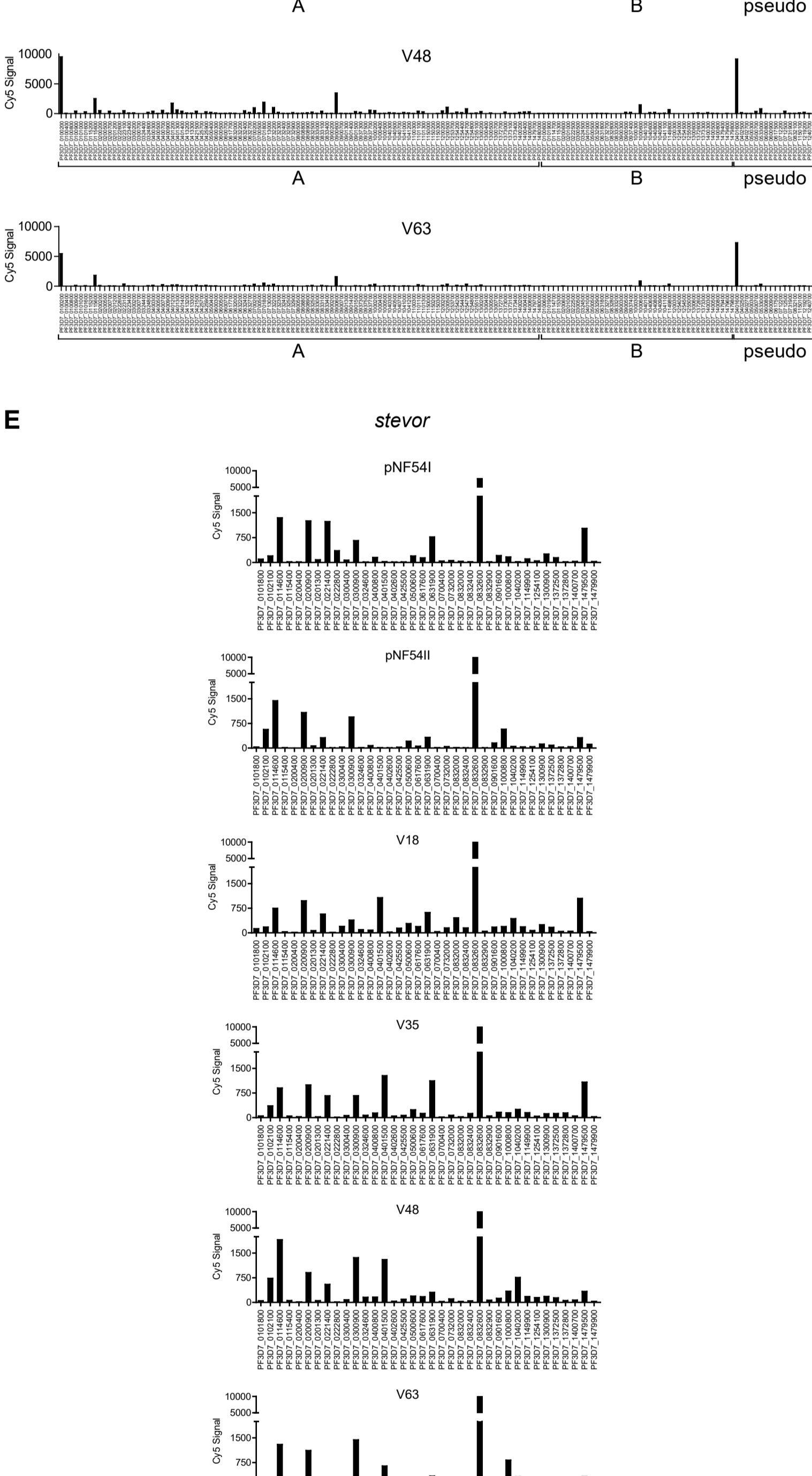

**E**

*stevor*

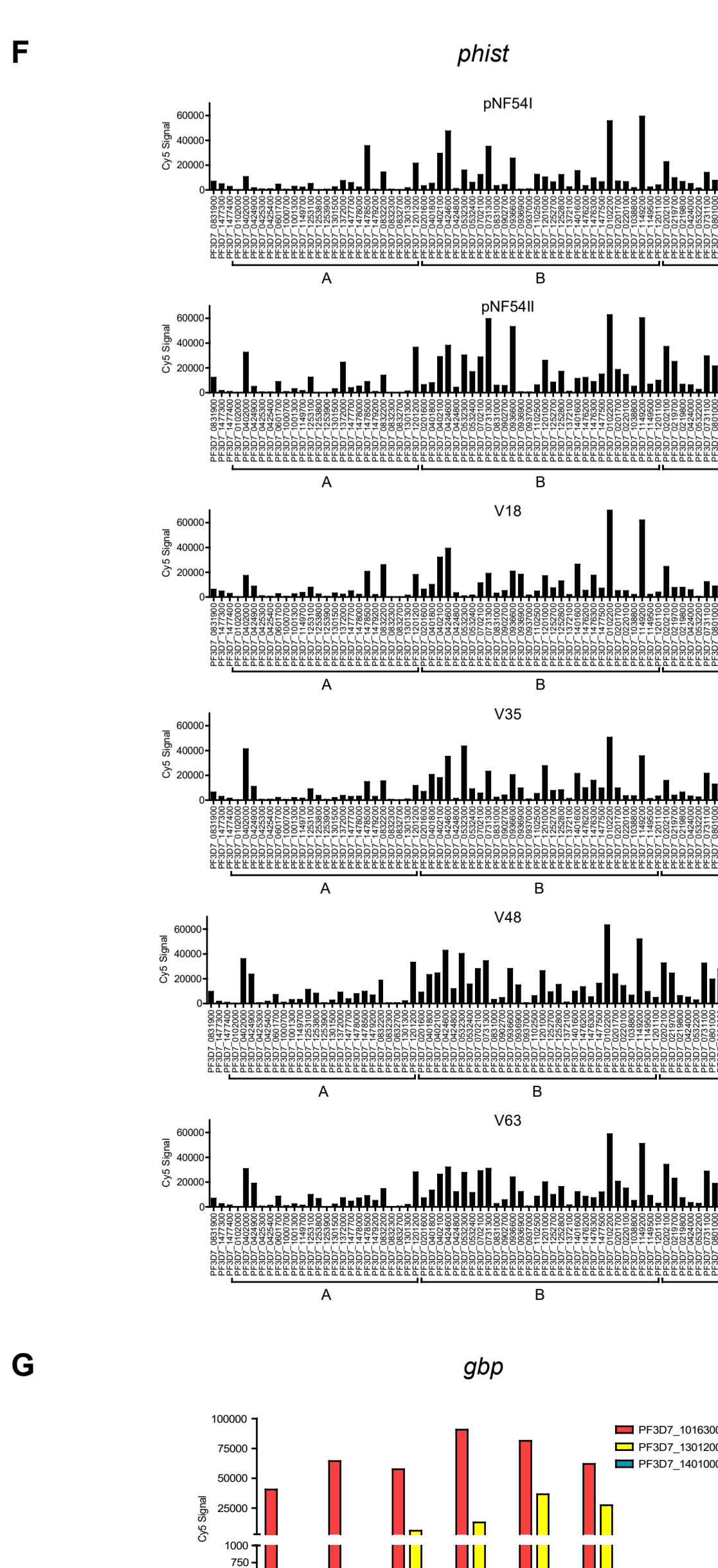

**F**

*phist*

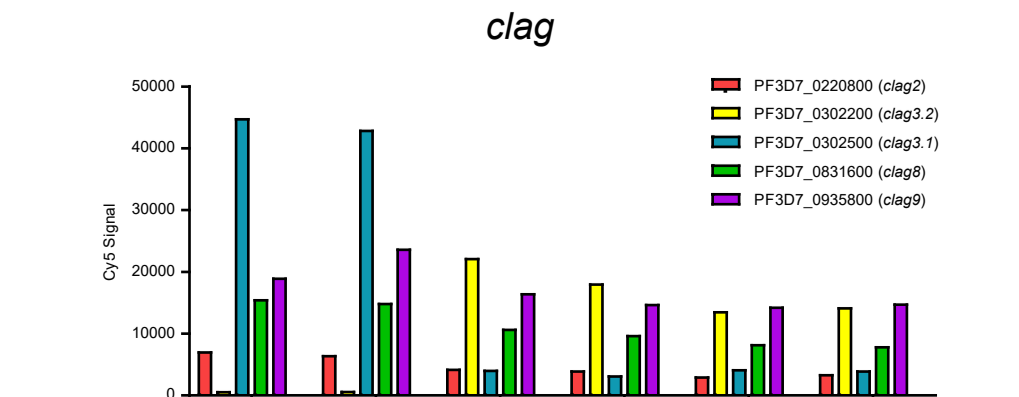

**G**

*gbp*



**H**

*clag*

Supplement: FIG S2 [file mbio.01636-21-sf002.pdf]

**A**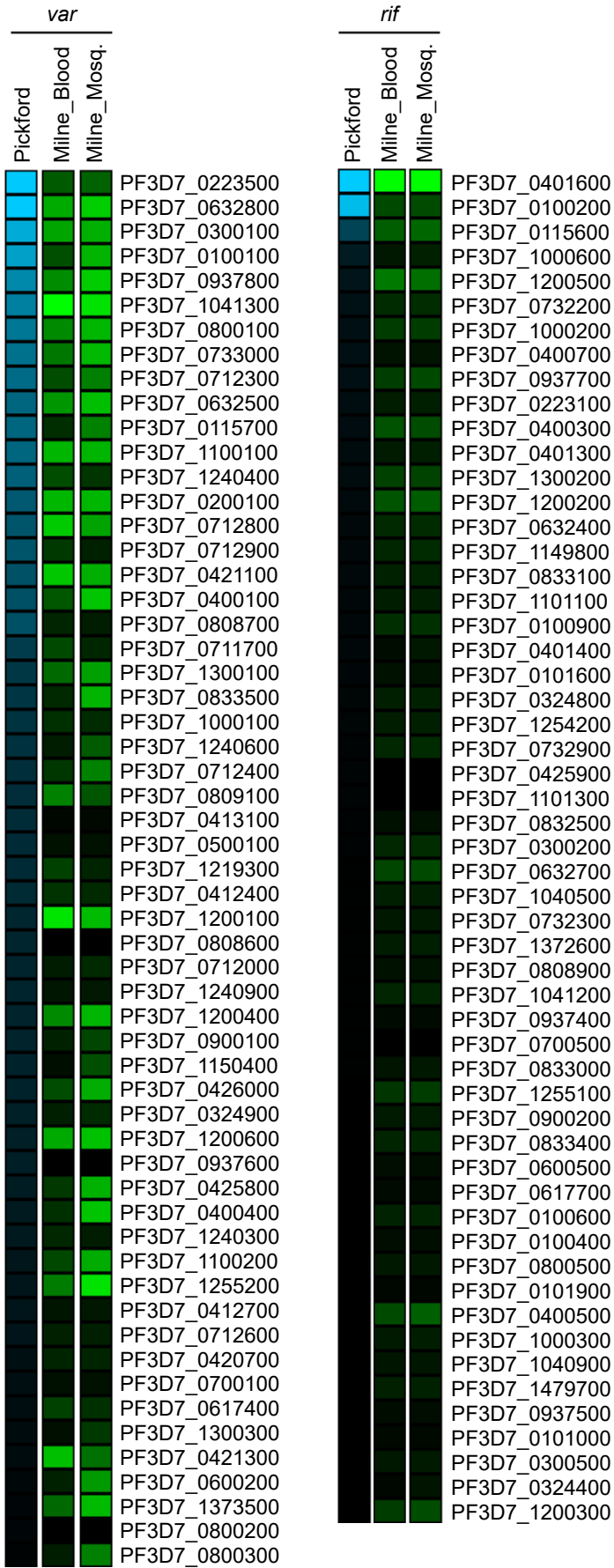**B**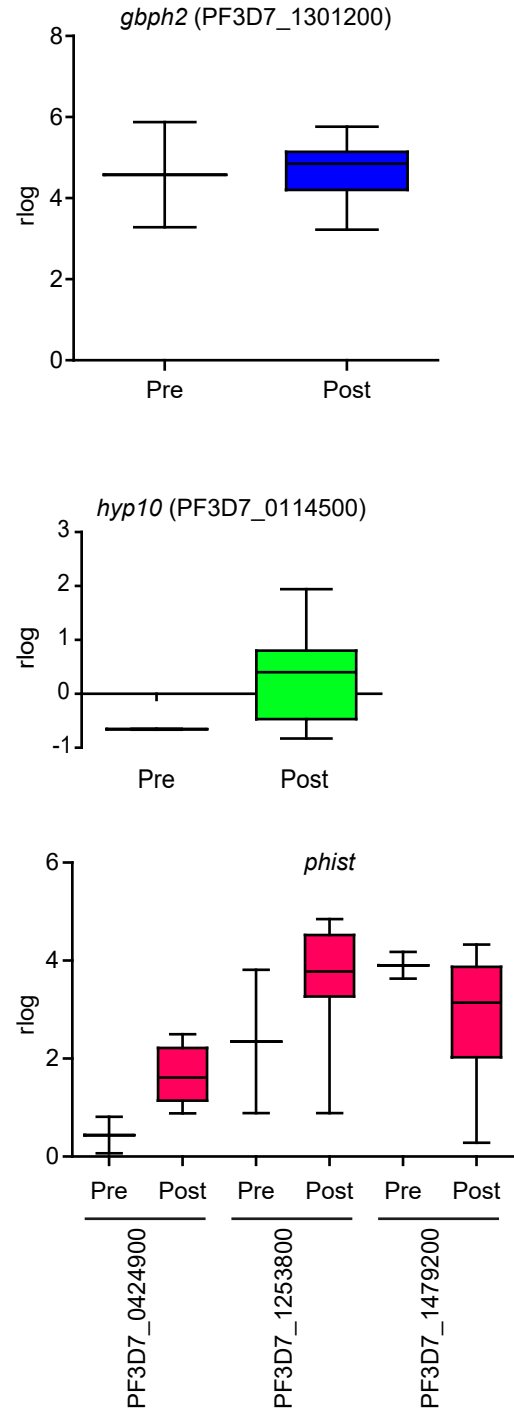**C**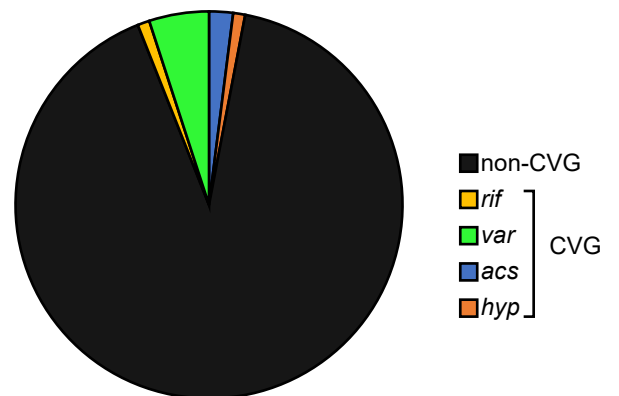

Supplement: FIG S3 [file mbio.01636-21-sf003.pdf]

# A

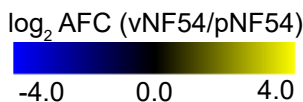

# B

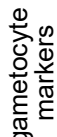

Supplement: FIG S4 [file mbio.01636-21-sf004.pdf]

**A**

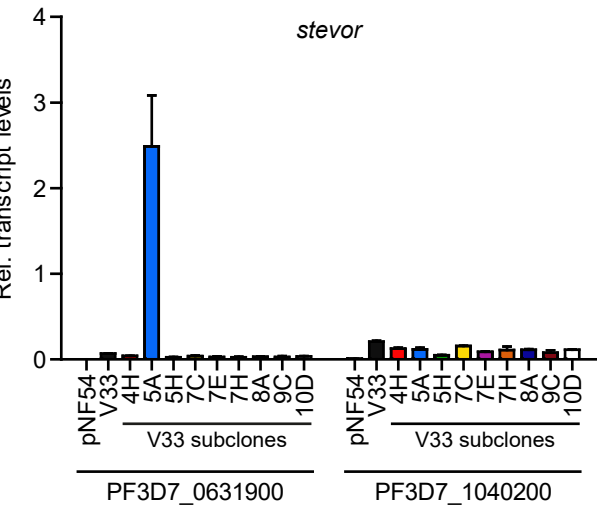

**B**

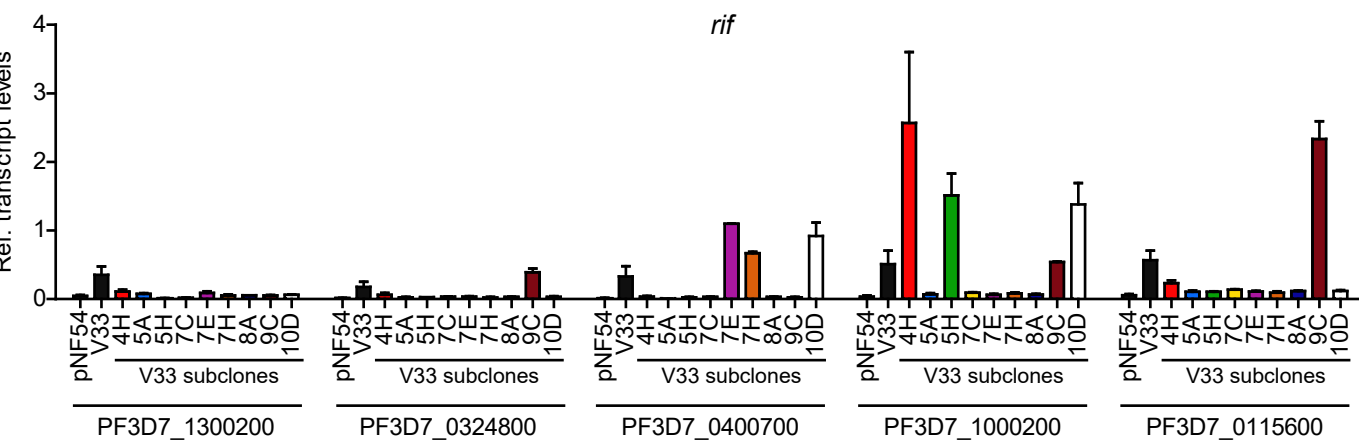

Supplement: FIG S5 [file mbio.01636-21-sf005.pdf]
